# Use of Group Quarantine in Ebola Control — Nigeria, 2014

**Published:** 2015-02-13

**Authors:** Cheri Grigg, Ndadilnasiya E. Waziri, Adebola T. Olayinka, John F. Vertefeuille

**Affiliations:** 1 Epidemic Intelligence Service, CDC; 2 Division of Healthcare Quality Promotion, National Center for Emerging and Zoonotic Infectious Diseases, CDC; 3 Nigeria Field Epidemiology and Laboratory Training Programme, Abuja; 4 Global Immunization Division, Center for Global Health, CDC

On July 20, 2014, the first known case of Ebola virus disease (Ebola) in Nigeria, in a traveler from Liberia (1), led to an outbreak that was successfully curtailed with infection control, contact tracing, isolation, and quarantine measures coordinated through an incident management system (2). During this outbreak, most contacts underwent home monitoring, which included instructions to stay home or to avoid crowded areas if staying home was not possible. However, for five contacts with high-risk exposures, group quarantine in an observation unit was preferred because the five had crowded home environments or occupations that could have resulted in a large number of community exposures if they developed Ebola.

On August 26, 2014, Nigerian authorities opened an observation unit in Lagos to function in conjunction with the Ebola isolation ward there. The observation unit housed the five quarantined asymptomatic contacts. The observation unit had eight beds in one large room, with four shared bathrooms. Additional living areas included a living room with a television and a kitchen with a microwave and refrigerator. Protocols developed for the unit required evaluation of each contact for clinical signs and symptoms of Ebola three times daily by the medical team. Quarantined contacts received instructions to avoid direct contact with each other and to avoid sharing items. Each contact was provided a pack of disinfecting wipes for use on common surfaces including door handles.

Visitors were restricted to the front porch of the unit, and food was delivered individually packaged with disposable utensils. An environmental health officer was stationed at the facility to disinfect the bathrooms after each use to minimize the potential for transmission between residents if one were to become infectious. Environmental health officers wore gloves, face masks, boots, scrubs, and aprons. Contacts housed in the unit were permitted to bring in personal items, including mobile telephones, with the understanding that if they developed symptoms of Ebola, their personal items would not be allowed to leave the facility.

Bringing exposed contacts together in group quarantine in an observation unit during an Ebola outbreak is not standard practice because the virus is only transmitted by exposure to body fluid when an infected person is symptomatic and because it is often not feasible to quarantine large populations of exposed persons in such facilities. Also, if one person in the observation unit becomes symptomatic, the 21-day observation period starts anew for each of the others based on their exposure to the newly symptomatic person. Alternatively, home monitoring of exposed, asymptomatic persons typically includes self-quarantine practices in conjunction with social distancing (i.e., avoiding crowded areas). Home monitoring of this sort minimizes both individual and public risk when effectively implemented.

In this instance, the five exposed persons could not be relied on to consistently adhere to social distancing nor to reliably report symptoms during home monitoring; thus, leaving them at home could have resulted in their coming into contact with large numbers of persons at their residence or workplace. Some resided in student dormitories, whereas others had public professions that required close contact with large numbers of persons. The observation unit allowed the contacts to be supervised to ensure that they did not come into contact with the general public, and that their health status was closely monitored.

Allowing the five identified contacts to stay in contact with the general public would have risked undermining containment efforts and spread of the virus to a third generation of patients. Before the observation unit opened, the contact tracing team had consistently maintained daily, in-person monitoring of >93% of all contacts, all of whom were traceable back to the person with the first recognized case of Ebola. The decision to use group quarantine versus home monitoring was made by balancing the practicalities of managing the observation unit effectively while simultaneously administering protocols within the observation unit to minimize risk to the persons housed there. Ultimately, none of the contacts quarantined in the observation unit developed signs of Ebola, and each of the persons were released at the conclusion of their individual, 21-day postexposure monitoring periods.

Lagos state had the resources to establish the observation unit and ensure that those observed were properly cared for. However, group quarantine of contacts in a central location might not be workable on a large scale.
